# Gastric cancer progression associated with local humoral immune responses

**DOI:** 10.1186/s12885-015-1858-9

**Published:** 2015-11-21

**Authors:** López-Vidal Yolanda, Ponce-de-León Sergio, Esquivel-Solís Hugo, Amieva-Fernández Rosa Isabel, Barreto-Zúñiga Rafael, Torre-Delgadillo Aldo, Castillo-Rojas Gonzalo

**Affiliations:** 1 Programa de Immunología Molecular Microbiana, Departamento de Microbiología y Parasitología, Facultad de Medicina, Universidad Nacional Autónoma de México (UNAM), Ciudad de México, Distrito Federal Mexico; 2 División de Investigación, Facultad de Medicina, Universidad Nacional Autónoma de México (UNAM), Ciudad de Mexico, Distrito Federal Mexico; 3 Dirección de enseñanza, Departamentos de Endoscopia y Gastroenterología, Instituto Nacional de Ciencias Médicas y Nutrición Salvador Zubirán (INCMNSZ), Ciudad de México, Distrito Federal Mexico; 4 Unidad de Biotecnología Médica y Farmacéutica, Centro de Investigación y Asistencia en Tecnología y Diseño del Estado de Jalisco, A.C., Guadalajara Jalisco, Mexico

**Keywords:** Gastric mucosa, Immune responses, Gastric cancer, IgG and IgA, *Helicobacter pylori*

## Abstract

**Background:**

Although the association between *H. pylori* and gastric cancer has been well described, the alterations studies are scarce in the humoral immune response in specific anatomical areas of stomach and during the stages of gastric cancer. The aim in this study was to determine the influence of humoral immune responses against *H. pylori* infection on gastric carcinoma.

**Methods:**

We selected 16 gastric cancer cases and approximately one matched control per case at the National Institute of Medical Sciences and Nutrition Salvador Zubirán (INCMNSZ); all the cases met the inclusion criteria for the study. We obtained three biopsies from each patient and from each of the predetermined regions of the stomach: *antrum*, *angular portion*, *corpus,* and *fundus*. From the patients with gastric cancer, additional biopsy specimens were obtained from tumor mid-lesion and tumor margin, and additional specimens were collected at least 2 and 5 cm from the tumor margin. We compared IgA levels against *H. pylori* in each area of stomach between cases and controls as well as between early and advanced stages of gastric cancer.

**Results:**

IgA values were strikingly elevated in cancer cases compared with control subjects; a value that was even higher in the distant periphery of tumor but was remarkably decreased toward the carcinoma lesion. The advanced stages of gastric cancer demonstrated the relapse of the humoral immune response in the mid-lesion region of the tumor compared with the tumor margins and adjacent non-tumor tissue.

**Conclusions:**

Gastric cancer is characterized by progressive accumulation of a concentrated, specific IgA response against *H. pylori,* beginning with an abnormal increase in the entire stomach but particularly in the adjacent non-tumor tissue. Thus, it is possible that this strong immune response also participates in some degree in the damage and in the development of gastric cancer to some extent.

## Background

*Helicobacter pylori* is a human pathogen that colonizes gastric mucosa and afflicts approximately half of the world’s population [1]. *H. pylori* infection is acquired mainly in the first years of life and persists for decades, causing chronic gastritis, duodenal ulcers, and gastric ulcers, and is a significant risk factor for the development of gastric adenocarcinoma [2]. The nature of gastroduodenal pathologies depends on the anatomical site of *H. pylori* infection in the stomach. We previously showed that the *antrum* and the *corpus* are the major anatomic sites colonized by *H. pylori* in patients with gastric cancer [3]. However, only a third of the gastric biopsies were positive for *H. pylori*, and its colonization was higher inside the tumor lesion compared with the surrounding non-tumor tissue. Therefore, it is tempting to speculate that vigorous abnormal immune responses at the local level are associated with the clearance of *H. pylori* infection and with gross pathology. Gastric adenocarcinoma develops as a consequence of chronic inflammation of the stomach lining caused by persistent infection with *H. pylori* [4]. Gastric carcinogenesis progresses through a sequence of preneoplastic lesions that manifest histologically as atrophic gastritis, intestinal metaplasia, and dysplasia [5]. Although a minority of infected people develops gastric cancer, this disease is the second leading cause of cancer death worldwide, partly because patients are not diagnosed until late-stage cancer is present and a poor prognosis [2].

*H. pylori* bacteria persist in spite of activation of the host’s innate and adaptive immune response [6]. Antibody production and cellular immune responses are not concordant with immunological memory against *H. pylori* infection [7]. Moreover, the bacteria seem to actively dampen the T-helper 1 (Th1) response, which is characterized by T cell activation (CD8 and CD4 positive T cells) and IFN-γ production, leading to considerable tissue damage [8, 9]. Factors other than *H. pylori* infection, which can predispose an individual to gastric cancer have been identified, among them are achlorhydria and oxyntic atrophy [10]. However, the relationship between gastric cancer development and the strength of local humoral immune responses against *H. pylori* is poorly understood.

IgA and IgG are the main effectors of the humoral immune responses against *H. pylori* infection in the gastric mucosa [4, 11, 12]. Unlike IgA, IgG is not actively secreted through the gastric mucosa; thus its protective function in the gastric lumen is limited [13]. Although IgA is actively secreted to the gastric lumen, where its effectors function is achieved, it is also present in the systemic circulation [14, 15]. Previous studies have shown that elevated serum levels of anti-*H. pylori* IgA is a sensitive indicator of gastric cancer risk [16, 17].

To determine the influence of humoral immune responses against *H. pylori* infection on gastric carcinoma, we assessed the presence of anti-*H. pylori* IgG and IgA levels in gastric adenocarcinoma patients and non-cancer patients by ELISA. We used tissue homogenates of different anatomical areas of the stomach and at the mid-lesion and marginal areas of the carcinoma lesion as well as nearby tumor-free tissue.

## Methods

### Patients and sampling

We used gastric samples from a previous study [3], in which patients underwent gastrointestinal endoscopy to rule out cancer and dyspeptic symptoms. Conducted between November 2006 and November 2007, the study included thirty-two patients recruited at the Endoscopic Service at the National Institute of Medical Sciences and Nutrition “Salvador Zubirán” (INCMNSZ). All samples were obtained with the written informed consent of the patients, given prior to their inclusion in the study and were in accordance with the Helsinki Declaration. This study was approved by the Ethics and Investigation Committee of National Institute of Medical Sciences and Nutrition “Salvador Zubirán”, registry number CIBH-1081. The subjects recruited were included in gastric cancer or control (non-cancer) groups; endoscopic diagnosis was confirmed by histological examination. A systematic biopsy-sampling scheme was used in order to obtained a maximum of three biopsies per patient from each predetermined regions of stomach: the *antrum, angular portion, corpus* and *fundus*. From the patients with possible gastric cancer, additional biopsy specimens were obtained from the mid-lesion of the tumor, the tumor margin, and at least 2 and 5 cm from the tumor margin. One part of each sample was snap-frozen and then stored at −70 °C until use. The other part was fixed in 10 % formalin and embedded in paraffin for histopathological examination. After diagnoses were confirmed, two patients in the cancer group were excluded from the study since they had MALT lymphoma. All patients with non-ulcer dyspepsia and/or gastro-esophageal reflux were considered the control group (non-cancer group). The groups were constituted as shown in Table 1.

**Table 1:** Characteristics of the study groups^a^

	Gastric cancer (*n* = 16)	Non-cancer (*n* = 14)
Mean age, yr (± SD)	57.6 ± 16.7	47.2 ± 13.3
Gender (Male/Female)	9/7	2/12
*H. pylori* colonization^b^	93.8 %*	64.3 %
Early stage of cancer (I and II)	5	-
Advanced stage of cancer (III and IV)	11	-
Positivity to *H. pylori* of each anatomic site		
* Fundus*	50	35.7
* Corpus*	56.2	50
* Angular portion*	50	35.7
* Antrum*	37.5	42.8
From the tumor^c^		
Mid-tumor	68.8	-
Tumor margin	68.8	-
At least 2 cm**	62.5	-
At least 5 cm**	56.3	-

^a^From our previous report [3]

^b^*H. pylori* was identified either by culture or PCR

^c^*H. pylori*-colonization in the tumor sites was 81.3 %

*p < 0.05

**At least 2 and 5 cm away from tumor margin

### Preparation of the strains to coated the ELISA plates

The *H. pylori* strains 26695 and J99 (ATCC 700392 and 700824, respectively) were growth on Casman agar (DIFCO) supplemented with 10 % defibrinated horse serum (Horse serum ATCC; Manassas, Va) and incubated at 36 ± 1 °C during 72 h in microaerophilic conditions. A Gram strain was performed to make sure that more than 90 % of the bacteria were bacilli. The strains were harvested in sterile isotonic saline solution (SISS), adjusted to 0.5 McFarland tube (1.5 × 10^8^ CFU/mL), and mixed 1:1 (*v/v*). The bacterial suspension was formalin treated with a 0.6 % formaldehyde solution v/v during 48 h at room temperature. Then, the bacterial suspension was centrifuged at 2,500 rpm and washed with SISS. This procedure was repeated once more; the bottom was re-suspended in SISS. The bacterial suspension was quantified by the Bradford method in order to know the concentration of the surface proteins.

### Measurement of *in situ* levels of IgG and IgA against *H. pylori*

We compared anti-*H. pylori* IgG and IgA levels, as measured by ELISA, in each region between cases and controls and in early (I and II) and advanced (III and IV) stages of gastric cancer. Each biopsy sample was homogenized individually in 50 μL of cold PBS (pH 7.4) using a glass tissue grinder on ice; the sample was subsequently adjusted to 500 μL. We took 100 μL of the sample and added 20 μL of a protease inhibitor cocktail (Complete, Roche Diagnostic GmbH, Mannheim, Germany) and 380 μL of 2 % saponin in PBS (JT Baker; Phillipsburg, NJ, USA). After overnight maceration at 4 °C, samples were centrifuged at 13,000 × g for 10 min. The protein concentration was determined and adjusted in all samples. One hundred μL of the supernatants were collected and individually tested for IgG and IgA levels by an indirect ELISA. Duplicate assay were performed for each immunoglobulin tested. The plates (NUNC MaxiSorp; Rochester, NY, USA) were coated with 100 μL of 10 μg/ml bacterial suspension in carbonate buffer pH 9.4 and incubated at 4 °C overnight. They washed three times with 0.5 % Tween 20 in phosphate buffered saline (PBS, pH 7.4). One hundred μL of each sample were inoculated in the ELISA plate and incubated for 1.5 h at room temperature. Antibodies (anti-human IgG and IgA, HRP-conjugated) were purchased from ZYMED Laboratories (Invitrogen; Carlsbad, CA, USA) and used at 1:5,000 for 1 h at 37 °C. After washing, substrate solution was added and the colorimetric reaction was stopped at 15 min. The ELISA plates were then read using a microplate reader (GENios Plus, Tecan Austria GmbH, Grödig, Austria). The results are given as the median ± interquartile range of the optical density values (450 nm) of each group. Positive and negative controls were serum obtained from patients of previous study [18].

### Determination of IgG1 and IgG2 antibody titer

96-well white polystyrene assay plates (Costar, Corning Inc., Lowell, MA, USA) were coated with 100 μL of 10 μg/ml bacterial suspension in carbonate buffer pH 9.4 and incubated at 4 °C overnight. The wells were then blocked with 5 % skim milk in PBS for 1 h, and then washed with 0.5 % Tween 20 in PBS. The patients’ sera were serially diluted in the wells and incubated for 1 h at 37 °C. After washing away the primary antibody, a mixture of secondary antibodies was added at final concentrations of 1:1,000 (α-IgG1) and 1:500 (α-IgG2). These secondary antibodies (α-IgG1, HRPO conjugated) were purchased from CALTAG Laboratories (Burlingame, CA, USA) and ZYMED (α-IgG2 AP conjugate, San Francisco, CA, USA). After 1 h of incubation, the plates were washed and Luminata Crescendo ELISA HPR substrates (Millipore Corporation, USA) were added sequentially to detect the IgG1 and IgG2 antibodies. The plates were then read using a microplate reader (GENios Plus, Tecan Austria GmbH, Grödig, Austria). The antibody titer was calculated by plotting luminescence *versus* serum dilution; the luminescence value was 1 above the background while the data were analysed by calculating the log10 of the titre dilution as described by Martinez-Becerra *et al.,* [19].

### Detection of *H. pylori* colonization

We have previously reported the *H. pylori* colonization status of the patients in this study [3]. We smeared an aliquot of the biopsy-homogenates previously described onto Casman agar plates (BBL Microbiology Systems, Cockeysville, MD, USA) for culture of *H. pylori*. Biopsies with negative culture were also tested by *16S rRNA* PCR according to Castillo-Rojas *et al.* [18]. Biopsies that were positive by culture or PCR were considered positive for *H. pylori* colonization. Biopsies were defined as *H. pylori*-negative if both results were negative.

### Statistical analysis

We had a maximum of three biopsy samples for each anatomical location. We obtained a single estimation for each biopsy location by calculating their arithmetic mean. Given that the tissue levels of immunoglobulin calculated in this way showed a right-skewed distribution, we used the median value as an index of central tendency and the interquartile range (IQR) to summarize the distribution. Patients with gastric cancer were divided into two subgroups according to their TNM stage: early gastric cancer subgroup for those in stage 0, I or II, and advanced gastric cancer subgroup for those in stage III or IV. Statistical comparisons of IgG and IgA levels were performed by the presence or absence of gastric cancer in the studied patients for each location as well as early and late stages of cancer in cancer patients through the rank sum test. We also tested the significance of differences between the predetermined locations, both in cancer and non-cancer patients, as well as between the tumor sampled sites in the cancer patients with the Friedman test (non-parametric procedure for a repeated measurements design with more than two repetitions per subject) [20]. The correlation of serum IgG with tissue IgG was measured with the Spearman’s rho coefficient. Given that, for each immunoglobulin, five different comparisons were performed, according to Bonferroni’s procedure, alpha value was adjusted to de 0.01 (two tailed) level. Calculations were performed with the Stata software (StataCorp. 2005. Stata Statistical Software: Release 9. College Station, TX: StataCorp LP).

## Results

Of the 30 patients that were recruited for the study, 16 were diagnosed with gastric cancer after endoscopic and histological examination, and the remaining 14 were positive for dyspepsia. A maximum of three biopsies of specific gastric anatomical sites were obtained per patient. As previously reported, sampled of gastric cancer patients were obtained from the tumor site, the tumor margin, and at least two and five centimeters beyond the margin [3]. We found that 64.3 % of the patients of the normal anatomical and 93.8 % of the gastric cancer patient areas were colonized by *H. pylori.* For their part, biopsies from the middle lesion, the margin, at least 2 and plus 5 cm, were colonized in 68.8, 68.8, 62.5 and 56.3 %, respectively. We found a discrete, but consistent relationship between the tissue dysplasia and the grade of colonization by *H. pylori* (Table 1). On the other hand, the rest of patients were negative for both the culture and PCR.

The results of tissue immunoglobulin assays in patients with and without gastric cancer are shown in Table 2. The more striking differences are evident in the significant IgA increase in the predetermined sites of stomach in the patients with gastric cancer compared with tumor-free patients (Optical Density median, IQR: *antrum* 0.868, 0.578–0.945 *vs*. 0.176, 0.129–0.867; *p* = NS; *angular portion* 0.802, 0.637–1.051 *vs*. 0.275, 0.135–0.945, *p* = NS; *corpus* 0.836, 0.688–1.039 *vs*. 0.413, 0.134–0.737, *p* = 0.006; *fundus* 0.772, 0.668–1.115 *vs*. 0.267, 0.160–0.675, *p* = NS). Additionally, it is clear that a differential distribution within tumor sites was observed in the patients with gastric cancer; the center samples showed the lowest values (Optical Density median, IQR: 0.419, 0.152–0.736) compared with the rest of the stomach (Optical Density median, IQR: tumor margin 0.902, 0.536–0.975; at least 2 cm 0.976, 0.606–1.220; at least 5 cm 0.919, 0.753–1.293), having significant differences (*p* = 0.001) even with the predetermined normal anatomical sites (*p* = 0.004). Although IgG levels were also higher in the anatomical sites of the stomach in patients with gastric cancer compared with controls, the differences were not statistically significant. However, the center of the tumor had the lowest IgG values (Optical Density median, IQR 0.193, 0.119–0.311) while higher levels were found farther away from the center of the tumor (Optical Density median, IQR: tumor margin 0.300, 0.138–0.463; at least 2 cm 0.276, 0.165–0.631; at least 5 cm 0.215, 0.164–0.445), a difference that was statistically significant (*p* = 0.005).

**Table 2:** Determination of IgA and IgG antibodies against *H. pylori* in gastric tissue by sampling site and presence of gastric cancer

Immunoglobulin A			
	Non-cancer *n* (14)	Gastric cancer *n* (16)	
Sampling site	OD Median (IQR)	OD Median (IQR)	*p* value*
Antrum	0.176 (0.129–0.867)	0.868 (0.578–0.945)	NS
Corpus	0.413 (0.134–0.737)	0.836 (0.688–1.039)	0.0068
Fundus	0.267 (0.160–0.675)	0.772 (0.668–1.115)	NS
Angular portion	0.275 (0.135–0.945)	0.802 (0.637–1.051)	NS
*p* value (predetermined sites)	NS	NS	
Mid-lesion	.	0.419 (0.152–0.736)	
Tumor margin	.	0.902 (0.536–0.975)	
At least 2 cm	.	0.976 (0.606–1.220)	
At least 5 cm	.	0.919 (0.753–1.293)	
*p* value (tumor sites)		0.001	
*p* value (predetermined sites + tumor sites)		0.0048	
Immunoglobulin G			
	Non-cancer *n* (14)	Gastric cancer *n* (16)	
Sampling site	OD Median (IQR)	OD Median (IQR)	*p* value*
Antrum	0.125 (0.107–0.615)	0.200 (0.160–0.375)	NS
Corpus	0.199 (0.115–0.326)	0.335 (0.226–0.627)	NS
Fundus	0.135 (0.116–0.462)	0.342 (0.161–0.527)	NS
Angular portion	0.151 (0.103–0.608)	0.247 (0.157–0.349)	NS
*p* value (predetermined sites)	NS	NS	
Mid-lesion	.	0.193 (0.119–0.311)	
Tumor margin	.	0.300 (0.138–0.463)	
At least 2 cm	.	0.276 (0.165–0.631)	
At least 5 cm	.	0.215 (0.164–0.445)	
*p* value (tumor sites)		0.005	
* p* value (predetermined sites + tumor sites)		NS	

*OD* Optical Density, *IQR* interquartile range

*Non-cancer *vs* Gastric cancer

*NS* not significant

When cancer patients were further divided into early (I and II) or advanced (III and IV) stages, differences were also found in immunoglobulin distribution across the tumor sites in the “advanced” gastric cancer patients (Table 3). The tumor center remained the site with the lowest values, both for IgG (Optical Density median, IQR 0.151, 0.103–0.233) and IgA (Optical Density median, IQR 0.273, 0.150–0.632). The comparison of patients in the early stage of gastric cancer with those without cancer was not statistically significant (*p* = 0.08 and *p* = 0.06 for IgA in the *antrum* and *corpus*, respectively), although the early gastric cancer was small (*n* = 5).

**Table 3:** Determination of IgA and AgG antibodies against *H. pylori* in gastric tissue from gastric cancer group by sampling site and cancer stage

Immunoglobulin A			
	Early gastric cancer *n* (5)	Advanced gastric cancer *n* (12)	
Sampling site	OD Median (IQR)	OD Median (IQR)	*p* value*
Antrum	0.913 (0.578–0.937)	0.825 (0.689–0.945)	NS
Corpus	0.870 (0.688–0.957)	0.823 (0.735–1.039)	NS
Fundus	0.668 (0.668–0.772)	0.851 (0.717–1.115)	NS
Angular portion	0.637 (0.637–0.833)	0.881 (0.744–1.075)	NS
*p* value (predetermined sites)	NS	NS	
Mid-lesion	0.811 (0.474–0.827)	0.273 (0.150–0.632)	NS
Tumor margin	0.941 (0.762–1.147)	0.889 (0.484–0.965)	NS
At least 2 cm	1.175 (0.606–1.220)	0.869 (0.628–1.225)	NS
At least 5 cm	0.919 (0.753–0.928)	0.915 (0.642–1.308)	NS
*p* value (tumor sites)	NS	0.0023	
*p* value (predetermined sites + tumor sites)	NS	0.0083	
Immunoglobulin G			
	Early gastric cancer *n* (5)	Advanced gastric cancer *n* (12)	
Sampling site	OD Median (IQR)	OD Median (IQR)	*p* value*
Antrum	0.375 (0.192–0.563)	0.182 (0.156–0.289)	NS
Corpus	0.473 (0.273–0.513)	0.285 (0.175–0.627)	NS
Fundus	0.516 (0.340–0.527)	0.299 (0.159–0.441)	NS
Angular portion	0.258 (0.236–0.550)	0.222 (0.142–0.348)	NS
*p* value (predetermined sites)	NS	NS	
Mid-lesion	0.285 (0.281–0.534)	0.151 (0.103–0.233)	NS
Tumor margin	0.508 (0.463–0.565)	0.221 (0.119–0.348)	NS
At least 2 cm	0.631 (0.296–0.926)	0.212 (0.133–0.501)	NS
At least 5 cm	0.439 (0.215–0.558)	0.188 (0.161–0.372)	NS
*p* value (tumor sites)	NS	0.0097	
*p* value (predetermined sites + tumor sites)	NS	NS	

*OD* Optical Density, *IQR* interquartile range

*Early gastric cancer *vs* Advanced gastric cancer

*NS* not significant

Serum determinations of IgG1 and IgG2 showed no difference in medians in both groups studied (data not shown). Correlation of serum and tissue immunoglobulins did not show a significant trend in either for the whole group or for the subgroups of presence of gastric cancer or of *H. pylori* (Table 4).

**Table 4:** Correlation of mean tissue IgG at predetermined sites with serum IgG 1 and 2^a^

Group	n	IgG1	*p* value	IgG2	*p* value
All	21	- 0.0753	NS	0.0492	NS
Non-cancer	10	- 0.1619	NS	0.2953	NS
Gastric cancer	11	- 0.0302	NS	- 0.0812	NS
*H. pylori*- negative	6	- 0.6473	NS	- 0.0588	NS
*H. pylori*-positive	17	0.2112	NS	0.1306	NS

^a^Spearman’s rho

*NS* not significant

## Discussion

The systematic analysis of IgA and IgG levels across different normal regions of the stomach (*antrum, angular portion, corpus,* and *fundus*), and the primary tumor and its surrounding tissue, allow us to define the dynamics of the humoral immune response against *H. pylori* and its association with the tissue pathology.

Topographic analysis showed that IgA levels were higher than IgG levels, except in the region of *corpus* where, coincidently, *H. pylori* colonized in higher frequency [3, 18]. Patients with gastric cancer had twice as much anti-*H. pylori* IgA than control patients, although IgG levels were similar in all patients. It is clear that the primary tumor expressed less IgA and IgG than the rest of the tissue, which had an abnormal higher production of IgA compared with controls. This increase in IgA secretion in the infected tissue correlates with previous studies that demonstrated serum IgA values might indicate gastric cancer risk development.

On the contrary, reduced levels in the center of the tumor led us to speculate that the damage to the tissue induced by chronic infection with *H. pylori* affects the production of immunoglobulin. Quiding-Järbrink *et al.,* [21] shown a decrease in IgA production in the stomach of patients with gastric cancer, suggesting that low levels of antibody production could be an indicative of risk for gastric cancer development in the case of precancerous atrophic gastritis caused by *H. pylori.* On the other hand, Adamsson *et al.,* [22] found reduced IgA levels in the non-tumor tissue from gastric cancer patients, suggested that this must be used as a marker for the detection of risk cancer group and early stage of gastric cancer. This study confirmed de early study of Quiding-Järbrink *et al.* Furthermore; we found similar results in the IgA antibody levels in the patients with GC as in the control group *H. pylori* positive (data not shown), as in Adamsson *et al.,* study [22].

Contrary our findings disagree with this previous reports in which cancer patients in various stages of gastric cancer progression were examined; in as much as more advanced cancers were associated with decreased antibody production. Conversely, we found an increase of IgA in the tissue of patients with gastric cancer, probably this is due by the changes in the *H. pylori* phenotypes during the development of gastric cancer, as we previous report [3], we found high genotypic diversity in the gastric cancer group. Quiding-Järbrink *et al.,* [21] suggested that a shift in antigens expression would probably lead to production of antibodies to the newly expressed antigens.

Previous studies have shown that IgA antibodies against *H. pylori* are detected in gastric tissue and saliva [23, 24]. Together with this data, we found a higher production of IgA in the tissue of patients with early and advanced gastric cancer stages compared with those patients without cancer. Then, detection of IgA antibodies against *H. pylori* in saliva should be increased at least two-fold. This will be a useful method to detected patients in early stages with increased risk of gastric cancer. This must be corroborating with a conducted study in future.

## Conclusions

In conclusion, gastric cancer is characterized by progressive accumulation of a concentrated, specific IgA response against *H. pylori,* beginning with an abnormal increase in the entire stomach but particularly in the adjacent non-tumor tissue. Thus, this strong immune response may also take part in the damage to some degree as suggested by the higher levels of humoral immune responses nearest to the tumor as compared to the adjacent normal tissue.

## Abbreviations

ELISA: Enzyme-linked immuno sorbent assay
IgA: Immunoglobulin A
IgG: Immunoglobulin G
IQR: Interquartile range
OD: Optical density
Th1: T-helper 1 response
